# Low Nitric Oxide Bioavailability Increases Renin Production in the Collecting Duct

**DOI:** 10.3389/fphys.2020.559341

**Published:** 2020-11-17

**Authors:** Andrew C. Curnow, Sabrina R. Gonsalez, Venkateswara R. Gogulamudi, Bruna Visniauskas, Eric E. Simon, Alexis A. Gonzalez, Dewan S. A. Majid, Lucienne S. Lara, Minolfa C. Prieto

**Affiliations:** ^1^Department of Physiology, Tulane University School of Medicine, New Orleans, LA, United States; ^2^Instituto de Ciências Biomédicas, Universidade Federal do Rio de Janeiro, Rio de Janeiro, Brazil; ^3^Department of Medicine, Tulane University School of Medicine, New Orleans, LA, United States; ^4^Instituto de Química, Pontificia Universidad Católica de Valparaíso, Valparaíso, Chile; ^5^Hypertension and Renal Center of Excellence, Tulane University School of Medicine, New Orleans, LA, United States

**Keywords:** eNOS knockout mice, cyclic GMP, L-NAME, NO donor, ODQ

## Abstract

In the kidney, the stimulation of renin production by the collecting duct (CD-renin) contributes to the development of hypertension. The CD is a major nephron segment for the synthesis of nitric oxide (NO), and low NO bioavailability in the renal medulla is associated with hypertension. However, it is unknown whether NO regulates renin production in the CD. To test the hypothesis that low intrarenal NO levels stimulate the production of CD-renin, we first examined renin expression in the distal nephron segments of CD-eNOS deficient mice. In these mice, specific CD-renin immunoreactivity was increased compared to wild-type littermates; however, juxtaglomerular (JG) renin was not altered. To further assess the intracellular mechanisms involved, we then treated M-1 cells with either 1 mM L-NAME (L-arginine analog), an inhibitor of NO synthase activity, or 1 mM NONOate, a NO donor. Both treatments increased intracellular renin protein levels in M-1 cells. However, only the inhibition of NOS with L-NAME stimulated renin synthesis and secretion as reflected by the increase in *Ren1C* transcript and renin protein levels in the extracellular media, respectively. In addition, NONOate induced a fast mobilization of cGMP and intracellular renin accumulation. These response was partially prevented by guanylyl cyclase inhibition with ODQ (1H-[1,2,4] oxadiazolo[4,3-a]quinoxalin-1]. Accumulation of intracellular renin was blocked by protein kinase G (PKG) and protein kinase C (PKC) inhibitors. Our data indicate that low NO bioavailability increases CD-renin synthesis and secretion, which may contribute to the activation of intrarenal renin angiotensin system.

## Introduction

The interactions of the renin-angiotensin system (RAS) with other hormonal systems to regulate blood pressure are complex. It is well accepted that the RAS is anti-natriuretic, and that nitric oxide (NO), bradykinin (BK), atrial natriuretic peptide, endothelin-1, and prostaglandins promote natriuresis. In the kidney, the activation of the RAS results in sodium retention and hypertension, independent from the systemic RAS (Kobori et al., 2003; Gonzalez-Villalobos et al., 2013). Renin, the rate-limiting step in the activation of the RAS cascade (Castrop et al., 2010), is synthesized and secreted by the granular juxtaglomerular (JG) cells. Physiologically, these processes are regulated by sympathetic innervation, renal perfusion pressure, and NaCl content in the macula densa cells (Castrop et al., 2010), and are modulated by other factors, such as NO (Campbell and Henrich, 1990; Chiu, 1996; Kurtz and Wagner, 1998; Persson, 2003). Nonetheless, studies on the role of NO in JG-renin are conflicting. *In vitro*, acute administration of NO donors inhibits renin secretion, while prolonged NO bioavailability stimulates it, suggesting that alternative intracellular pathways are at play (Kurtz et al., 1998).

In the collecting duct (CD), during pathophysiological conditions, the principal cells produce renin. This activates the intrarenal and intratubular RAS and increases angiotensin II (Ang II) formation (Prieto-Carrasquero et al., 2004; Gonzalez and Prieto, 2015a,b). In mice, the augmented urinary excretion of the renin substrate, angiotensinogen (AGT), positively correlates with renal Ang II concentration and increases systolic blood pressure, but not plasma Ang II (Kurtz and Wagner, 1998; Kobori et al., 2002, 2003), supporting the concept that intratubular RAS activation contributes to the pathogenesis of hypertension. In contrast to JG-renin, Ang II acts in a feed-forward fashion to stimulate CD-renin synthesis and secretion via Ang II type 1 receptor (AT1R) through a Ca^2+^/PKCα-dependent augmentation of cAMP and activation of PKA/CREB pathway (Gonzalez et al., 2011; Lara et al., 2017). Furthermore, the activation of the BK/B2 receptor (B2R) increases CD-renin synthesis and secretion through PKC stimulation and NO release in mouse CD cells (Lara et al., 2017). In that study, we observed that L-NAME, an L-arginine analog that inhibits NO synthase (NOS) activity, completely blocked BK/B2R-stimulated CD-renin. However, L-NAME, *per se*, increased CD-renin synthesis (Lara et al., 2017). This observation suggests that in pathophysiological conditions associated with decreases in NO bioavailability, as occurs in hypertension (Kobori et al., 2003), CD-renin is augmented and contributes to intrarenal RAS activation. Thus, we propose that fine-tune control of intrarenal NO levels strictly regulates CD-renin synthesis and secretion. In the present study, we tested the hypothesis that low intrarenal NO levels stimulate the production of CD-renin. To address this hypothesis, we first used kidneys from mice with cell-type specific deletion of eNOS in the CD (CD-eNOS-KO) to examine renin immunoexpression status during NOS deficiency. Then, we used M-1 cells to assess the intracellular signaling involved in the NO-dependent regulation of CD-renin. Our data indicated that the lack of NO bioavailability enhances CD-renin synthesis and secretion, and is a possible mechanism that contributes to L-NAME- or NO-knockout hypertensive mice models. However, augmented NO levels using NO donors promote CD-renin intracellular accumulation, but not renin secretion, which may contribute to decreased intrarenal and intratubular RAS activation as a renal protective mechanism.

## Materials and Methods

### Renin Immunohistochemical Studies in Kidney Samples From CD-eNOS-KO Mice

Kidney sections (3 μm) from homozygous endothelial nitric oxide synthase (eNOS)-deficient male mice (Jackson Laboratory, Bar Harbor, ME, United States) were obtained as collaboration from E.E.S [(Tulane University HSC, Department of Medicine, Nephrology (Altaf-M et al., 2013)]. Immunohistochemical studies were performed using immunoperoxidase technique, as previously described (Prieto-Carrasquero et al., 2004, 2008). Three kidney sections per mouse (CD-eNOS-KO - *N* = 5; Wild-type - *N* = 5) were sequentially incubated with (1) normal blocking rabbit serum, primary antibody (rabbit renin polyclonal antibody generously provided by Dr. Tadashi Inagami, Vanderbilt University) at 1:8,000 concentration, (2) biotin-conjugated rabbit anti-mouse secondary antibody, (3) avidin-biotinylated horseradish peroxidase H complex (ABC Elite Vectastain; Vector Laboratories Inc., Burlingame, CA, United States), and (4) DAB (0.1% 3,3′-diaminobenzidine tetrahydrochloride, Sigma, St. Louis, MO, United States) to visualize peroxidase activity. From each mouse kidney section (3 μm), an average of 20 microphotographs using 20 × objective was captured using a digital camera. The intensity of renin immunostaining was analyzed using the NIS Elements software from Nikon. An average of these values was expressed as arbitrary units ± SE.

### Pharmacological Tools and Antibodies Used in the “*in vitro*” Study

Diethylamine NONOate (NO donor) was purchased from Enzo Life Sciences (Cat # ALX-430-014-M005, Farmingdale, NY, United States). L-NAME (NOS inhibitor, Cat # N5751), Calphostin C (protein kinase C – PKC – inhibitor, Cat # 20785), and ODQ (soluble guanylyl cyclase inhibitor, 1H-(1,2,4)oxadiazolo[4,3-a]quinoxalin-1-one, Cat # O3636) were purchased from Sigma-Aldrich (Saint Louis, MO). A selective protein kinase G (PKG) inhibitor, KT5823, was purchased from TOCRIS (Cat # 1289, Bristol, United Kingdom).

We used a mouse anti-renin polyclonal IgG B-12 antibody (sc-81178) for detection of renin, and ß-actin was detected using a goat anti- ß-actin monoclonal IgG antibody (sc-1615), both purchased from Santa Cruz Biotechnology, (Santa Cruz, CA, United States). For western blot procedures the secondary antibodies used were the IR Dye 800CW anti-goat and mouse according to the primary antibody chosen (Li-Cor Bioscience, NE, United States). For immunofluorescence, we utilized a mouse anti-renin polyclonal IgG H-105 antibody (sc-22752–1:200), and secondary Alexa Fluor antibodies (Alexa fluor-488), both purchased from Life Technologies (Carlsbad, CA, United States). The mouse kidney cortical CD cells (M-1 cell line) were purchased from American Type Culture Collection (Cat: CRL-2038, ATCC, Manassas, VA, United States).

### Cell Culture With Experimental Design

The M-1 cell line has many CD-like characteristics, including epithelial morphology and CD-specific antigens, and is composed of primarily principal and intercalated cells (Stoos et al., 1991). M-1 cells were cultured as previously described (Gonzalez et al., 2016). Three experimental groups were used in this study: (1) CTRL: control (PBS, pH 7.4), (2) L-NAME: incubation with 1 mM N(ω)-nitro-L-arginine methyl ester (L-NAME - NOS inhibitor), and (3) NONOate: incubation with 1 mM diethylamine NONOate (NONOate - NO donor). In all conditions, incubation time was 8 h. To detect the involvement of key signaling enzymes in the NO-mediated intracellular mechanism, the following were added to the cell culture media before NONOate incubation: (1) Calphostin C, a PKC inhibitor (10 nM), (2) KT5823, a PKG inhibitor (10 nM), and (3) ODQ (10 mM), the soluble guanylyl cyclase (GC). The phosphodiesterase inhibitor, 3-isobutyl-1-methylxanthine (IBMX, 1 mM Cat: I5879 Sigma-Aldrich) was used for cGMP preservation prior to assay. We used 5–6 different sets of cell cultures (control and experimental groups; *n* = 6) for each experimental design mentioned above.

### RNA Isolation and Quantitative Real-Time RT-PCR (qRT-PCR)

RNA extraction was performed using a commercially available kit (Qiagen, Hilden, Germany). To evaluate the renin gene expression, qRT-PCR was performed using the TaqMan PCR system as previously described (Lara et al., 2012), and the data obtained were normalized to β-actin mRNA expression levels. Primers (1 uM) and probes (900 nM) used to amplify renin mRNA were: Forward: 5′-AGT-ACT-ATG-GTG-AGA-TCG-GCA-TT-3′, Reverse; 5′-AGA-TTC -ACA-ACC-TCT-ATG-ACT-CCT-C-3′, and fluorogenic probe: 5′6-FAM-TTC-AAA-GTC-ATC-TTT-GAC-ACG-GGT-TCA-G- BHQ1-3. The mouse β-actin gene was used as an internal standard: Forward: 5′-ATC-ATG- AAG-TGT-GAC-GTT-GA-3′, Reverse: 5′-GAT-CTT-CAT-GGT-GCT-AGG-AGC-3′, and fluorogenic probe: 5′-6-HEX-TCT-ATG-CCA-ACA-CAG-TGC-TGT-CTG-GT-BHQ2-3′. Results are presented as a ratio between the levels of mRNA of the interest gene against β-actin.

### Protein Detection and Quantification

For western blot analysis, the cells were lysed in a buffer containing 1 mmol/L EDTA, 20 mmol/L HEPES–Tris (pH 7.0), 250 mmol/L sucrose, 25 μL/mL of protease inhibitor cocktail (Sigma-Aldrich), and 30 μL/mL of phosphatase inhibitors. Mechanical lysing was done with culture dish scrapers, and aspiration and release with a 31G needle and syringe for three times was utilized to homogenize the content. Subsequently, the sample homogenates were centrifuged, and the supernatant collected. Protein concentration was determined by a bicinchoninic acid protein assay kit (Pierce, Rockford, IL, United States).

### Immunofluorescence in M-1 Cells

Under identical conditions as the experimental groups mentioned above, M-1 cells were grown in chamber slides (Nunc Lab-Tek Chamber Slide System, Sigma-Aldrich). The cells were fixed with 4% paraformaldehyde and blocked (Image-iT FX signal enhancer, Invitrogen, Carlsbad, CA, United States). The cells were incubated with a mouse anti-renin antibody (1:200) overnight at 4°C, and with its respective green anti-mouse secondary antibody (1:1000 - Alexa fluor 488) for 1 h. ProLong Gold antifade reagent containing 4′,6-diamidino-2-phenylindole (Invitrogen, Carlsbad, CA, United States) was used as a nuclear stain. Digital images were captured from three fields from three different sets of cell cultures using 40× magnification (Nikon Eclipse 50i fluorescence microscope).

### Quantification of cGMP Levels

For cGMP quantification cells were lysed in 0.1 M HCl and stored with IBMX as described above. The cGMP levels of M-1 cells were determined using an ELISA kit (cat #581021, Cayman, Ann Arbor, MI, United States) according to the manufacturer’s instructions.

### Quantification of Renin Content in the Cell Culture Media

The renin amount was determined in cell culture media by using a kit from Molecular Innovations (Mouse prorenin/renin total antigen assay – Cat: MPRENKT-TOT, Novi, MI, United States). Cell culture media was concentrated 100 × using Amicon Ultra Centrifugal Filters 0.5 mL – Ultracell 10 KDa (Cat: UFC501096, Merck-Millipore, Burlington, MA, United States) before the assay.

### Statistical Analysis

Data were expressed as mean ± SEM. Statistical differences were assessed by Student’s *t* test or one-way ANOVA with Dunnet’s post-test. Significance was defined as *P* < 0.05. Statistical analysis was performed using GraphPad Prism 8.0 (San Diego, CA, United States).

## Results

### CD-eNOS-KO Mice Exhibit Augmented Immunoexpression of CD-Renin

**FIGURE 1:**
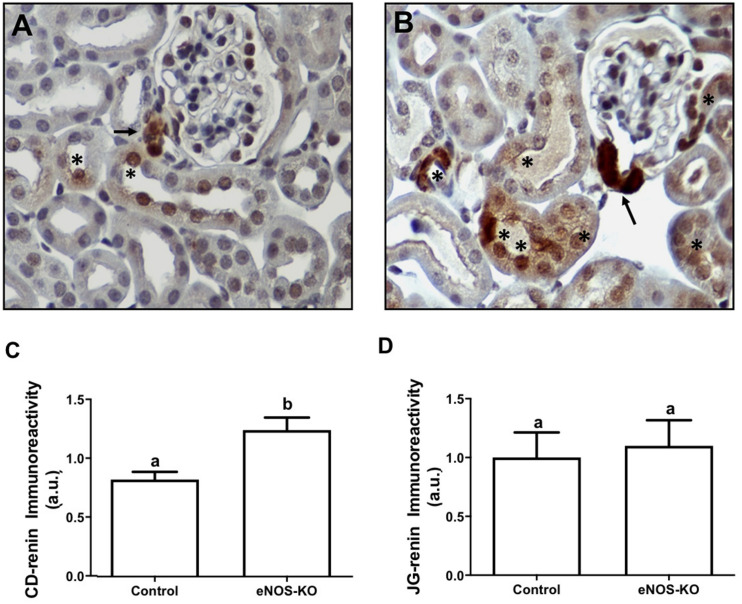
CD renin is augmented in mice with NOS deficiency in the collecting duct. Immunohistochemistry was performed as described in the Materials and Methods section. Representative digital images from kidneys of wild type (*n* = 5) **(A)** and *CDeNOS-KO* (*n* = 5) **(B)** mice taken, respectively at 400 × magnifications show specific renin staining (brown) in the juxtaglomerular (JG) cells (arrows) and in the CD cells (asteristic). **(C)** Quantification of brown staining in the collecting duct from wild type and *CDeNOS-KO* mice (*P* = 0.0006). **(D)** Quantification of brown staining in the JGA cells from wild type and *CDeNOS-KO* mice (*P* = 0.7619). Different letters represent statistical significance that was defined as *P* < 0.05 compared to CTRL (student’s *t*-test).

To assess if the eNOS gene disruption alters CD-renin immunoexpression, we used the immunoperoxidase technique to examine specific renin immunostaining in paraffin embedded kidney (3 μm) sections of control and CD-eNOS-KO mice. Figure 1 shows microphotographs of kidney sections from control and CD-eNOS-KO mice, demonstrating increased specific renin immunoexpression in the CD from CD-eNOS-KO mouse kidneys (40 × objective; Figures 1A,B, respectively). CD-renin immunostaining quantification (Figure 1C) was 45% higher in the kidneys of CD-eNOS-KO mice as compared to controls (*P* = 0.0006). There was no statistical difference noted in JG-renin from the CD-eNOS-KO when compared to controls (Figure 1D).

### Local NO Production Affects Renin Synthesis and Protein Content in M-1 Cells

**FIGURE 2:**
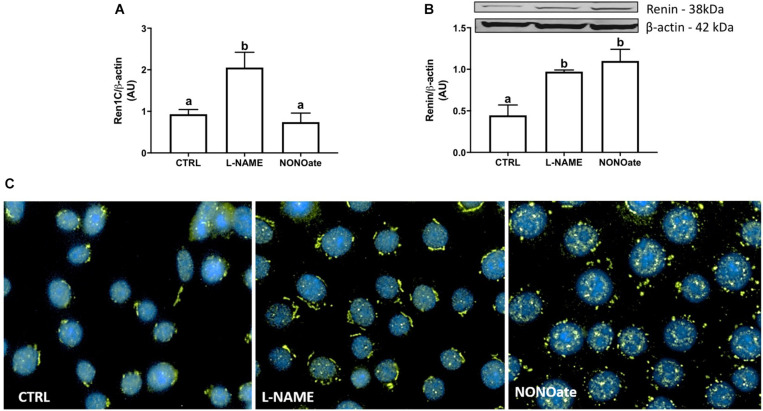
NO bioavailability stimulates renin expression in M-1 cells. **(A)** qRT-PCR amplification of M-1 cells renin (*Ren1C*) gene. M-1 cells were incubated with 1 mM L-NAME (NOS inhibitor) or 1 mM NONOate (NO donor) for 8 h in parallel to the control (CTRL). Results were expressed as mean ± SE in arbitrary unities (AU) (*P* < 0.0001). **(B)** Upper panel – representative image of renin protein (∼ 40 kDa band) detection by Western blot. Lower panel – Densitometric analysis of renin band normalized against β-actin densitometry. Results were expressed as mean ± SEM in arbitrary units (AU). Different letters represent statistical significance (*P* < 0.0001; one-way ANOVA followed by Dunnet’s post-test). **(C)** Immunofluorescence of renin (green) expression in M-1 cells incubated with 1 mM L-NAME or with 1 mM NONOate. Representative images were obtained using a 40× objective.

First, we examined the *Ren1C* gene expression in M-1 cells by qRT-PCR. Treatment of cultured M-1 cells with L-NAME for 8 h increased renin transcript from 0.93 ± 0.05 (CTRL) to 2.05 ± 0.15 (*n* = 6, *P* < 0.0001; Figure 2A). The increase in *Ren1C* gene expression directly increased renin synthesis, as renin protein content detected by the quantification of the 38 KDa band was doubled compared to the control (0.97 ± 0.01 vs. 0.45 ± 0.06, *n* = 4, *P* = 0.0002; Figure 2B). The incubation with the NO donor, NONOate, did not change renin mRNA expression (Figure 2A), but also increased the renin protein content (1.10 ± 0.07 vs. 0.45 ± 0.06, *n* = 4, *P* = 0.0004; Figure 2B). The same profile was observed in immunofluorescence of M-1 cells: higher detection of renin, marked in green, in cells incubated with either L-NAME or NONOate, when compared to CTRL (Figure 2C).

### Deficient NO Bioavailability Augments Renin Secretion in M-1 Cells

**FIGURE 3:**
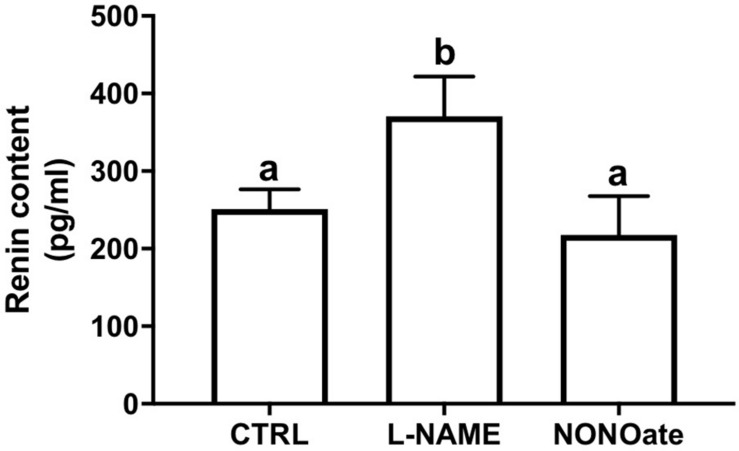
Lack of NO bioavailability stimulates renin secretion in M-1 cells. Renin content in the cell culture media after incubation with 1 mM L-NAME or 1 mM NONOate. Results were expressed as mean ± SE in pg/mL. Different letters represent statistical significance *P* = 0.0011 compared to control, CTRL (one-way ANOVA followed by Dunnet’s post-test).

The M-1 cells were incubated with L-NAME or NONOate, and renin content in the cell culture media was quantified using a renin-specific ELISA kit. Under these conditions, renin content was higher in the media from the cells incubated with L-NAME compared to the control (370 ± 25 vs. 251 ± 11 pg/ml, *n* = 5, *P* = 0.001; Fig. 3). Incubation with NONOate did not change the renin amount in the extracellular media compared to the control (NONOate: 218 ± 25 pg/ml, *n* = 5; Figure 3).

### NONOate Intracellular Signaling Involving CD-Renin Accumulation

**FIGURE 4:**
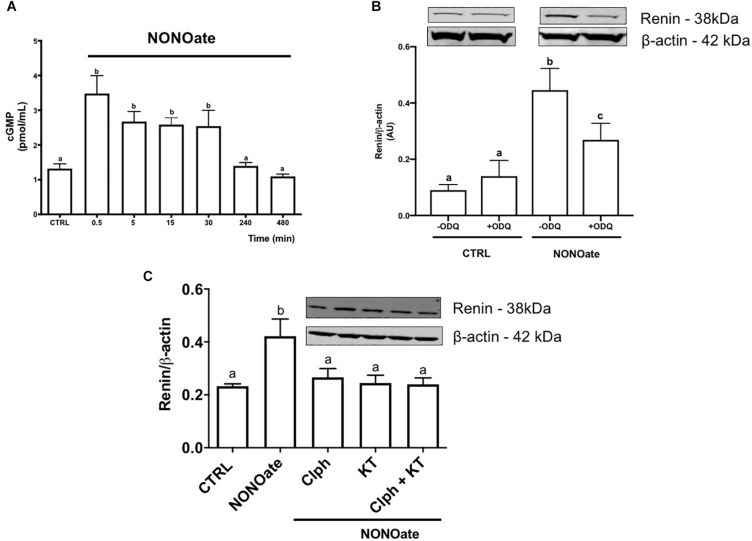
NONOate induces intracellular renin accumulation through activation of sGC/cGMP/PKG and PKC pathways. **(A)** Time-course cGMP intracellular mobilization (in pmol/mL) in the presence of 1 mM NONOate during 8 h. Results were expressed as mean ± SE. (0,5 min *P* < 0.0001; 5 and 10 min *P* = 0.0069; 30 min *P* = 0.0069; 240 min *P* = 0.9997, and 480 min *P* = 0.9499 comparing to control, CTRL). **(B)** M-1 cells were incubated or not with 10 mM guanylyl cyclase inhibitor ODQ in the absence or in the presence of 1 mM NONOate. Results were expressed as mean ± SE. (CTRL in the absence of ODQ vs. CTRL in the presence of ODQ *P* = 0.0472; CTRL vs. NONOate, both without ODQ *P* = 0.00001; CTRL vs. NONOate in the presence of ODQ *P* = 0.0042). **(C)** M-1 cells were incubated with 1 mM NONOate in the absence or in the presence of 10 nM KT5823 (a PKG inhibitor) or/and 10 nM Calphostin C (Clph, a PKC inhibitor). Upper panels display renin protein (38 kDa band) detected by Western blot. Lower panels show densitometric analysis of renin band normalized against β-actin. Results were expressed as mean ± SE in arbitrary units (AU). Different letters represent statistical significance (CTRL vs. NONOate *P* = 0.0049; CTRL vs. NONOate + Clph *P* = 0.8776; CTRL vs. NONOate + KT5823 *P* = 0.9968; NONOate + Clph P + KT5823 = 0.9997, one-way ANOVA followed by Dunnet’s post-test).

To determine whether M1-cells are able to mobilize cGMP, a time-course quantification of the cGMP levels in the presence of NONOate. The intracellular NONOate-dependent cGMP production was fast, with a maximal effect in the first 30 s of incubation. At 30 s, cGMP content was 2-fold higher in the cells incubated with NONOate as compared to the control (3.48 ± 0.51 vs. 1.32 ± 0.14, *n* = 6, *P* < 0.0001; Figure 4A). The cGMP levels remained statistically elevated compared to the control until 30 min after incubation. Longer incubation periods with NONOate (longer than 4 h), resulted in cGMP levels that were unchanged from basal levels. Concomitant treatment with NONOate and ODQ, a GC inhibitor, reduced intracellular renin amount by 35% compared with NONOate alone (0.27 ± 0.034 vs. 0.45 ± 0.045 arbitrary units, *n* = 3, *P* = 0.03). No difference was found with ODQ alone compared with the control (0.11 ± 0.01 vs. 0.09 ± 0.01, *n* = 3, *P* = 0.19; Figure 4B). Furthermore, NONOate-mediated intracellular accumulation of CD-renin was totally blocked by PKG inhibition with KT5823 (0.25 ± 0.06 vs. 0.42 ± 0.03, *n* = 3, *P* = 0.011; Figure 4C). Equally important, PKC inhibition with Calphostin C prevented NONOate-mediated stimulation of intracellular CD-renin (0.27 ± 0.03 vs. 0.42 ± 0.03, *n* = 3, *P* = 0.027; Figure 4C). Concomitant treatment with KT5823 and Calphostin C exerted similar effects as each inhibitor alone (Figure 4C).

## Discussion

The present study demonstrates that a lack of intracellular NO bioavailability in the collecting duct increases CD-renin synthesis and secretion, as revealed by the distal nephron segments of CD-eNOS-KO mice demonstrating augmented renin protein immunoexpression and M-1 cells treated with L-NAME (NOS inhibitor) showing increased CD-renin synthesis and secretion. Surprisingly, increased NO availability, through incubation with NONOate, also resulted in accumulation of intracellular CD-renin, although secretion of renin appeared to be suppressed. Incubation of NONOate activates the cGMP/GC/PKG pathway and PKC-dependent intracellular renin accumulation. Our data indicate that in the CD, low NO levels stimulate CD-renin synthesis and secretion, thus leading to an inappropriate intratubular RAS activation, which may contribute to the development and progression of hypertension. On the other hand, CD-renin secretion does not occur with treatment with NO donors.

Mice with global eNOS deficiency exhibit increased blood pressure, decreased heart rate (Shesely et al., 1996), and augmented susceptibility to develop kidney damage (Khan et al., 2013). In the kidney, NOS activity is approximately 25-fold greater in the medulla than the cortex (Wu et al., 1999). The greatest sources of NO within the renal medullary region are the collecting ducts and the vasa recta (Wu et al., 1999). The production of NO in the inner medullary collecting ducts contributes to an acute pressure-natriuresis response by buffering reactive oxygen species (ROS). During low endogenous NO production, the augmentation of medullary oxidative stress reduces blood flow, Na^+^ retention, and the development of hypertension (Cowley et al., 2015). Giani et al. (2014) demonstrated that in L-NAME induced hypertension there is low NO bioavailability and augmentation of Ang II, particularly in the renal medulla, and that these responses are attenuated in mice lacking renal ACE. It is likely that reduced local NO in the renal inner medulla further contributes to the upregulation of renin production by the principal cells of the CD, and ultimately intrarenal Ang II content. In collecting duct nitric oxide synthase 1β knockout (CDNOS1KO) mice under changes in dietary salt, CD renin does not change in renal medullary tissues (Hyndman et al., 2017). However, in CD-eNOS-deficient mice used in the present study, renin immunostaining in cortical and medullary renal CD cells was augmented but not in JG cells. However, we cannot exclude the role of nNOS – which is also abundantly present in the CD (Roczniak et al., 1998; Hyndman et al., 2013) – or iNOS (Choi et al., 2012) and eNOS isoforms from adjacent cells (Heeringa et al., 1998), in the local NO production and regulation of CD-renin. The role of eNOS in the pathogenesis of hypertension is evident as its deficiency provokes a similar magnitude of hypertension as observed in the triple n/i/eNOS null transgenic mouse model (Morishita et al., 2005; Tsutsui et al., 2015). The expression of eNOS also decreases in the kidney during hypertension (Yang et al., 1998; Paek et al., 2000). Moreover, following unclipping of the renal artery in the two-kidney, one-clip (2K1C) rat Goldblatt model of experimental hypertension, there was an associated restoration of eNOS expression (Paek et al., 2000).

In M-1 cells, augmented NO bioavailability increased renin protein intracellularly. To dissect the intracellular mechanism by which NO regulates CD-renin, we used NONOate, as a NO donor, and L-NAME, as a NOS inhibitor. However, treatment with NONOate did not affect CD-renin synthesis. Rather, NONOate induced intracellular renin accumulation without altering *Ren1C* gene expression. The ribosome-associated protein 1 (RACK1) interacts with PKC-ßII affecting protein but not the transcript (Gallo and Manfrini, 2015). In mice, RACK1 activates translational changes downstream of PKC without affecting mRNA levels (Volta et al., 2013). It is possible that NONOate activates PKC via RACK1; therefore, increases in renin content might be due to PKC phosphorylation of RACK1. Further studies are required to define these aspects.

An open question is how NONOate did not increase renin secretion. Multiphoton studies demonstrate that prorenin (the vast majority) and renin seems to be secreted toward both the tubular lumen and the interstitium because of the high density of quinacrine granules visualized at both the apical and basal regions of the CD principal cells (Kang et al., 2008). It has been shown, in the cardiovascular system, exogenous NO inhibits exocytosis of endothelial granules by S-nitrosylation of N-ethylmaleimide sensitive factor (NSF), a key component of the exocytic machinery (Lowenstein, 2007). It is plausible to speculate that the same occurs in the renin granules of the CD.

In M-1 cells, the fast-intracellular cGMP mobilization was also associated with intracellular renin accumulation, as ODQ partially negated the effect of NONOate. In JG-renin, the direct role of NO remains controversial and dependent on the signaling pathway activated. NO either enhances JG-renin secretion by inhibition of cAMP degradation (Kurtz et al., 1998; Kashiwagi et al., 2000; Castrop et al., 2010) or inhibits JG-renin secretion through cGMP activation (Castrop et al., 2010). In JG cells, cyclic-GMP-dependent protein kinases (PKG) are predominantly found in association with renin storage granules or the plasma membrane (Kurtz and Wagner, 1999). It was demonstrated that PKG acts as an antagonist of cyclic-AMP-stimulated JG renin secretion (Kurtz and Wagner, 1998). Because ODQ partially prevented the accumulation of renin protein in M-1 cells, we propose that NO may act via cGMP-dependent and independent pathways. Dey et al. (2009) demonstrated that cGMP leads to down-regulation of PKG-I via the ubiquitin-proteasome pathway. This is a possible ODQ -mediated mechanism leading to repress NONOate-dependent intracellular accumulation of renin. Taking together, the underline mechanism in the stimulation of intracellular renin accumulation may involve the NO/GC/cGMP/PKG pathway. Because Calphostin C inhibits NO-dependent intracellular accumulation of renin, we propose that PKC is also involved in the regulation of renin in collecting duct cells. The lack of additional effects when PKG and PKC inhibitors are used in combination indicates that these kinases act on the same pathway.

Treatment with L-NAME stimulates both renin expression and renin secretion. It is worth highlighting that L-NAME inhibits all three isoforms of NOS; thus L-NAME- induced responses cannot be interpreted as the involvement of eNOS only, as demonstrated in the CD-eNOS-KO mouse model. Accordingly, we previously showed that BK stimulates CD-renin synthesis and secretion via the B2R, and the subsequent PKC and NO pathways (Lara et al., 2017). Independent of renin localization, either in JG or principal cells of the CD, its synthesis is mediated by phosphorylation of the transcriptional factor CRE-binding protein (CREB) (Gomez et al., 2014; Gonzalez et al., 2015). Although, cAMP/PKA is the main signaling pathway involved in CREB phosphorylation, PKC can also phosphorylate CREB (Brindle and Montminy, 1992). However, we demonstrated that in the CD, the PKC pathway plays a major effect on the activation of renin synthesis (Gonzalez et al., 2015, 2017; Lara et al., 2017).

The impact of NO bioavailability on intracellular renin accumulation in M-1 cell cultures may explain animal models of hypertension. In the same model used in this work, ANG II infusion (25 ng/min, 14 days) display hypertension and kidney injury (Whiting et al., 2013), thus indicating that changes in CD-renin may impact intrarenal Ang II formation contributing to the development of hypertension. Furthermore, there are other studies that support our hypothesis: (1) Consensus about the role for nitrate and nitrite as antihypertensive molecules that act via NO formation-dependent and independent mechanisms and how nitrate/nitrite inhibit cardiovascular remodeling in hypertension (Guimarães et al., 2020). (2) The effects associated with the formation of NO and other NO-related molecules, may induce S-nitrosylation of target proteins. NO inhibits exocytosis of endothelial granules by S-nitrosylation of N-ethylmaleimide sensitive factor (NSF), a key component of the exocytic machinery (Lowenstein, 2007). This mechanism may explain how NONOate did not increase renin secretion. (3) Singh et al. (2017) proposed that increase in NO production helps to mitigate salt-sensitive hypertension induced by Ang II. (4) Hyndman et al. (2017) further demonstrated that low NO local bioavailability, as observed in mice lacking CD NOS1β, upregulates intra renal Ang II levels during high salt intake. In consequence, increased renal Ang II may also contribute to the inappropriate preservation of sodium reabsorption. Accordingly, augmentation of NO bioavailability contributes to reduce the development of hypertension due to decreased CD-renin secretion.

Currently, the role of (pro)renin on the stimulation of distal Na^+^ reabsorption in hypertension is being actively investigated, particularly due to the presence of the (pro)renin receptor (PRR) in the CD (Advani et al., 2009; Gonzalez et al., 2011; Prieto et al., 2017). Several studies indicate that the activation of the PRR by local (pro)renin regulate blood pressure via ENaC (Lu et al., 2016; Quadri and Siragy, 2016; Peng et al., 2017; Prieto et al., 2017; Quadri et al., 2018). Indeed, chronic Ang II-infusion in mice with specific PRR deficiency in the CD display attenuated blood pressure associated with decreases in fractional Na^+^ excretion, and reduced amounts of Ang II and renin in urine (Prieto et al., 2017). Furthermore, treatment of rats with PRR shRNA reduces the expression levels of α-ENaC throughout the kidney (Quadri and Siragy, 2016). These findings are intriguing because in the distal nephron segments, the PRR is mostly expressed in the intercalated cells and, to lesser extend in the principal cells. Although, the PRR in the principal cells seems to be enough for the regulation of (pro)renin-mediated stimulation of Na^+^ reabsorption via ENaC (Ramkumar et al., 2018), the underlying molecular mechanisms involved remains unclear. Whether the activation of PRR by (pro)renin, but not renin, solely stimulates ENaC activity (Lu et al., 2016) is a relevant functional issue that requires further investigation. Equally important, the soluble form of the PRR (sPRR), contributes to the activation of ENaC and chronically stimulates α-ENaC expression in cultured CD cells (Wang et al., 2020). The physiological relevance of this mechanism has been pointed out in pregnant rats. The administration of the PRR decoy inhibitor (PRO20) attenuates pregnancy-induced α-ENaC upregulation, Na^+^-water retention, and plasma volume expansion associated with suppressed intrarenal RAAS (Fu et al., 2019). In fact, sPRR acutely stimulates ENaC activity via Nox4-derived ROS (Wang et al., 2020). Taken together, we propose that the secretion of (pro)renin by the CD during low NO bioavailability further contributes to the stimulation of ENaC-mediated Na^+^ reabsorption.

**FIGURE 5:**
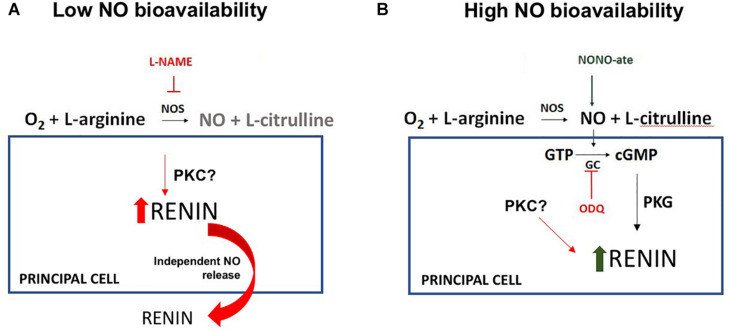
Proposed model for the NO-dependent molecular mechanism of regulation of renin in the collecting duct (CD-renin). **(A)** In the lack of NO production there is an increase in renin expression and secretion with a possible involvement of protein kinase C (PKC). **(B)** In the presence of high NO bioavailability, there is an increase in intracellular cGMP and protein kinase G (PKG) activation that is involved in a fast renin maturation. There is no renin secretion which contributes to the intracellular CD-renin accumulation.

In conclusion, our data indicate that reduced NO bioavailability enhances CD-renin synthesis and secretion; while augmented NO levels stimulate CD-renin intracellular accumulation. L-NAME induced renin secretion is evoked by a cGMP independent pathway (Figure 5). The direct *in vivo* effects of NO-mediated CD-renin accumulation remain difficult to interpret. It is possible that acute changes in NO modulate physiologic changes in CD-renin, while states of chronically suppressed or deficient NO contribute to sustained hypertension, although this requires further investigation. The imbalance between NO and RAS may promote augmented intrarenal Ang II levels that activates a feedforward mechanism of CD-renin synthesis and secretion mediated by AT1R/cAMP/PKA and Ca^+2^-dependent PKC-α (Gonzalez et al., 2015). Thus, we propose that decreased NO bioavailability, as seen in many forms of chronic kidney diseases, induces intrarenal RAS activation by stimulating CD-renin production, resulting in increased sodium reabsorption, hypertension, and kidney injury.

## Data Availability Statement

The raw data supporting the conclusions of this article will be made available by the authors, without undue reservation.

## Ethics Statement

The animal study was reviewed and approved by Tulane Institutional Animal Care and Use Committee.

## Author Contributions

VG and BV: Experimental design and performance and data analyses. ES: Provided mouse model with a cell-type specific deletion of eNOS in the CD. DM: Interpretation of the data. AG and LL: Supervision of the project, interpretation the data and manuscript and figures design and manuscript’s revisions. MP: Conception of the original idea, supervision of the project, interpretation of data and critical suggestions to the manuscript. All authors discussed the results and final version of the manuscript.

## Conflict of Interest

The authors declare that the research was conducted in the absence of any commercial or financial relationships that could be construed as a potential conflict of interest.
